# Economic Effects of Renewable Energy Expansion Policy: Computable General Equilibrium Analysis for Korea

**DOI:** 10.3390/ijerph17134762

**Published:** 2020-07-02

**Authors:** Inha Oh, Wang-Jin Yoo, Kihwan Kim

**Affiliations:** 1 Department of Advanced Industry Fusion, Konkuk University, Seoul 05029, Korea; inhaoh@konkuk.ac.kr; 2 Department of Industrial Engineering, Konkuk University, Seoul 05029, Korea; 3 Korea Energy Economics Institute, Ulsan 44543, Korea; kkihwan@keei.re.kr

**Keywords:** renewable energy, emission trading scheme, computable general equilibrium, solar photovoltaic, wind power, Korea

## Abstract

This study examines the effects of renewable energy expansion policy on the Korean economy and industries using the computable general equilibrium model, which divides the power generation sector into detailed generation technologies and sources. The scenarios are set to observe the cases where the share of solar photovoltaic and wind power generation reaches 7%. The effects are examined according to differing circumstances, such as when greenhouse gas (GHG) emissions are regulated, and the funding source for renewable expansion varies. The results show that renewable expansion policies have negative effects on GDP. However, the magnitude of the GDP decline becomes smaller when GHG emissions are regulated. The expansion of renewable energy induces the growth of upstream industries which supply components for renewable generation modules. Regarding employment, the renewable expansion policy can increase the demand for labor. However, the direction and the extent of the effect vary depending on the funding source. When overlapping regulations, such as the emission trading scheme and renewable energy expansion policies, exist in the power generation sector, the renewable energy expansion policy could provide incentives for GHG emission-intensive power sources.

## 1. Introduction

The Republic of Korea (henceforth “Korea”) must urgently take steps to reduce its greenhouse gas (GHG) emissions to cope with climate change. CO_2_ emissions in 2018 were 2.6 times those in 1990, making Korea the eighth largest CO_2_-emitting country in the world in 2018 [1]. Although Korea pledged to the international community to reduce GHG emissions by 37%, as compared to Business-As-Usual (BAU), by 2030 under the Paris Agreement [2], no reduction has occurred. On the contrary, Korea’s CO_2_ emissions increased at an annual average of 2.8% between 2015 and 2018 [1]. One aspect showing the inadequate efforts in coping with climate change in Korea is the minimal level of renewable energy utilization. According to the OECD [3], the share of renewable energy in the total primary energy supply in Korea was only 1.9% in 2018, which was below the OECD average of 10.5% and the lowest among the OECD countries.

The Korean government announced the “Renewable Energy 3020” plan in 2017 to promote renewable energy. It sought to increase the share of renewable energy in power generation to 20% by 2030 [4,5,6]. The plan included large-scale investments in solar photovoltaic (PV), wind power generation facilities, and related government support, which would add 30.8 GW (16.5 GW) of solar PV (wind) power generation capacity by 2030. The Korean government implemented the plan not only to respond to climate change but also to provide an economic stimulus to reduce unemployment and secure future growth engines [7,8]. Additionally, if the expansion of renewable energy leads to a decrease in air pollutant emissions, there may be benefits in terms of public health. The air pollution problem in Korea is very severe. In 2017, the mean population exposure to particulate matter with a diameter of 2.5 μm or less (PM_2.5_) was 25.1 μg/m^3^ for Korea, which was highest among all OECD member countries [9]. Exposure to high concentrations of PM_2.5_ increases the likelihood of lung and vascular diseases and increases the mortality rate [10]. In the case of Korea, about 37% of domestic PM_2.5_ emissions (primary and secondary) came from coal use (mostly coal power generation and the steel industry), about 37% came from oil use (mostly the transportation sector), and only 4% came from gas use (mostly gas power generation and household use) in 2014 [11]. PM_2.5_ emissions from gas power generation are much lower than those of coal power generation. If the expansion of renewable energy leads to a decrease in coal power generation, it is expected that there will be large benefits in terms of public health. On the other hand, if the expansion of renewable energy mainly replaces gas generation, the health benefits may be relatively smaller.

However, it is difficult to find studies that examine the environmental aspects as well as the effects of increased renewable energy on the Korean economy. Yun et al. [12] and Hwang et al. [13] examined the effects of changes in the generation mix on the economy but did not analyze the effects of renewable energy policies.

This study examines the effects of increased renewable energy power generation on the Korean economy using the computable general equilibrium (CGE) model. In particular, the CGE model developed for this study utilizes the newly developed input-output table for renewable energy in Korea [14], which divides the renewable energy sector into detailed classifications for each renewable generation technology. Hence, it is possible to observe the effects of policies regarding the specific renewable generation technologies (e.g., the expansion of solar PV power) on the economy as well as on each industrial sector.

The CGE model used in this study is a single country model that assumes a small open economy; it is a static model that assumes the myopic predictive ability of economic entities. The base year is 2015, which is the year that Korea’s latest input-output table was made available.

The scenarios used in this study are as follows: First, scenarios on the expansion of the shares of solar PV and wind power generation from the base year economy are observed. The same scenarios are observed again when there are regulations, such as the emission trading scheme (ETS), on GHG emissions. In the model, the increase in the renewable energy share is to be achieved via government financial support. Thus, scenarios are added to compare the case where the necessary fund for increasing renewable energy is financed with a lump sum tax to the case where the fund is financed via taxation of the power generation sector and the case where the fund is financed via levying a carbon tax on fossil fuel usage (as in [15]).

The composition of this study is as follows: Section 2 examines the existing literature that analyzes the effects of renewable energy expansion policies using the CGE model. Section 3 describes the structure of the model and the scenarios employed in this study. Section 4 describes the results of the analyses. Section 5 concludes and provides future research directions.

## 2. Studies on Analyzing the Effects of Renewable Energy Expansion Policies Using CGE Modelling

This section reviews the studies that analyzed the effects of renewable energy expansion policy using the CGE model [15,16,17,18,19,20,21].

Böhringer and Löschel [16] observed the effectiveness of the EU’s renewable energy initiatives. The model simulates the power generation technology in detail (divided into coal, petroleum, gas, nuclear power, biomass, wind power, hydropower, and solar power). The scenarios assumed that the share of renewable energy reaches 30% of the total power generation by 2020. The policy was financed by a lump-sum tax. According to the results, by 2020, the share of coal power generation decreased from 21.5% to 17%, while the share of gas generation decreased from 24% to 19%. However, the share of wind power generation increased from 5% to 14%, while the share of biomass generation increased from 2% to 5%. The total amount of government financing support was to reach 13.7% of the total electricity cost. And the total amount of power generation would increase through the financial support to the power sector, with electricity prices projected to decrease by 2.3%. The carbon emission showed decrease by 2.6%.

Böhringer et al. [17] analyzed the effect of renewable energy support on employment through the Feed-In-Tariff (FIT) policy in Ontario, Canada. The scenario used for analysis assumed that, on average, 22.3 ¢/kWh was supported for renewable energy development. Moreover, 47% of renewable energy generation parts would be procured domestically. As a result, electricity prices would rise by 13% and the demand for electricity would decline by 2.1%. Household income would reduce by 0.54%. Employment in the renewable energy sector increased significantly, creating about 12,400 jobs. However, it was more than offset by the decline of employment in other industries (fossil fuel power generation and energy intensive industries); thus, the overall unemployment rate increased by 0.32% (1.97 existing jobs would be eliminated for each new job created).

Böhringer et al. [15] analyzed the effect of Germany’s renewable energy policy. The rigidity of the labor market was assumed to closely examine the effect on employment. Various scenarios were created according to the funding source for policy implementation (a lump-sum tax, labor tax increase, electricity price increase, and reduced subsidy for coal power generation). When the policy was implemented, since renewable energy generation is labor-intensive, the demand for labor in the power generation sector increased. However, the magnitude of the effect varied greatly depending on the funding source and the extent of support.

Qi et al. [19] observed the effects on energy demand and GHG emissions when China achieves the renewable energy target (replacing 15% of total energy demand with non-fossil fuel by 2020). Thus, solar PV, wind, and biomass power generation capacities were to increase by about 62.5 times, 6 times, and 5.4 times, respectively, relative to 2010. The scenarios were developed using prospects on economic growth, extents of policy implementation, and the costs of renewable energies. According to the results, the GHG intensity of the Chinese economy decreased by only 2% through the policy. The low level of decrease could be attributed to the channel of carbon leakage since the government’s support resulted in a decline in the price of electricity, which resulted in the increased use of electricity. This, in turn, resulted in an increase in the use of fossil fuels in the power generation industry.

Dai et al. [20] estimated the economic and environmental impacts of China’s policies to introduce large-scale renewable energy projects. The model divided the capital stock into two parts: general capital and capital for non-fossil fuel power generation. The scenario assumed large investments in renewable generation capacities, to achieve wind, solar PV, and biomass power of 2000 GW, 2500 GW, and 600 GW, respectively, by 2040. About 9% of the country’s total investment in 2040 was to be invested in the non-fossil fuel generation sector. In 2050, the share of non-fossil fuel power generation was to reach 78%. However, the GDP decreased by only 0.3%. Additionally, about 4.12 million jobs would be created in 2050 in related upstream industries. The GHG emissions were projected to peak in 2025 and to decrease afterward.

Mu et al. [21] observed the impact of China’s renewable energy policy on employment. They observed employment regarding the production and maintenance of renewable energy generators, as well as employment induced by changes in electricity prices. According to the results, employment increased as solar PV and wind power generation increased, but these effects were temporary and were largely offset by the decrease in employment induced by the increased tax burden and electricity price.

Thus, the studies that focus on Europe (especially Germany) and China, which pursued aggressive renewable energy policies, have been examined. These studies used the CGE model to analyze the impact of support policies on the overall economy and impacts on key outcomes are summarized in Table 1. It is observed that most of the CGE models tried to elaborate on the power sector using data detailed by respective power generation technologies and sources. The effect on the overall economy was usually negative measured in terms of GDP and welfare, due to the large investments required. However, the extent of the decline in GDP varied depending on the extent of the policy and funding sources (e.g., funding for implementing policies such as FIT). It is generally accepted that the increase in the share of renewable energy generation will have a positive effect on the environment (in terms of GHG emissions reduction). Most studies addressed the impact on employment because governments expect that the implementation of renewable energy support policies would have positive effects on employment. However, the effect on employment vary depending on the direct and indirect factors affecting employment. The demand for labor may increase when the production of renewable energy generation devices increases. However, the demand for labor may decrease when electricity prices rise or the demand for labor drops in the existing power generation sectors. Additionally, the demand for labor may decrease, given the lowered investment and consumption in other areas of the economy.

## 3. Model Structure and Scenario

### 3.1. Outline of the Model

This section describes the model structure and the scenarios. The CGE model developed for analysis is a small open-economy of the Korean economy. The model is a static model that assumes myopic predictive ability of economic entities. Since the static model is utilized in this study, it is not possible to observe the transitional dynamics of the renewable energy expansion over time. This model instead focuses on long-run impacts of the renewable expansion policies. Hence, the results are comparing the situation when the policies are fully implemented to the situation at the base year economy. Böhringer et al. [17] also utilized a static model to observe the effects of full build-out of the renewable expansion policies, which the Ontario government expects to occur between about 2018 and 2020, using 2005 as a base year. In the static model, the economic agents are assumed to have a myopic foresight since their decisions are based on static expectations on the prices of goods. However, in the dynamic intertemporal model, the agents are assumed to have a perfect foresight and intertemporally optimizing their behavior using the price information on the future [22].

**Figure 1:**
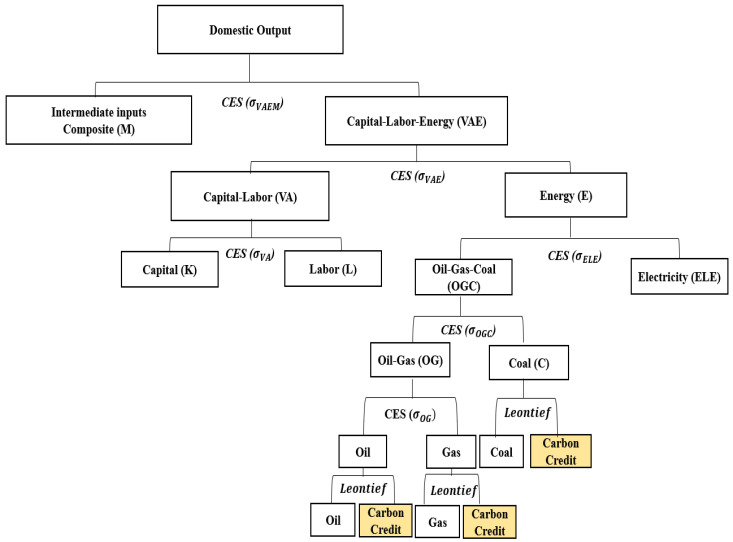
Nested production structure of industries (except the electricity sector).

Labor and capital are considered as the primary factors of production, which are employed along with energy and material inputs to produce domestic output. Thus, nested and separable “constant elasticity of substitution (CES)” production functions are employed to represent the substitution possibilities among capital, labor, energy, and material inputs (Figure 1).

Production of commodities is represented by the nested CES production functions, as shown in Figure 1. At the first level, a composite of intermediate material inputs (M) is traded off against an aggregate of energy, capital, and labor (VAE), subject to CES. At the second level, a CES function captures the substitution possibilities between the energy aggregate (E) and a value-added composite (VA) of labor and capital. At the third level, capital and labor substitution possibilities within the value-added composite are described by a CES function, whereas various energy inputs (coal, gas, oil, and electricity) enter the energy composite, subject to CES. Values for substitution elasticities between production factors of each sector are shown in Table 2 [23].

The final consumption demand is determined by the representative household, which maximizes welfare, subject to its budget constraint consisting of net factor income. The government collects taxes and transfers part of the income to the representative household and uses the rest for the government spending. The consumption demand of the representative agent is comprised of energy and non-energy consumption goods. In the model, the investment and provision of public goods and services are given exogenously. Additionally, if the ETS is implemented in the production structure, as represented in Figure 1, fossil fuel inputs (e.g., coal, gas, and oil) are combined with carbon credit (emission allowances) by the Leontief function. In proportion to the amount of fossil fuel (and its carbon contents and GHG emissions) inputs, carbon credits are required, reflecting the emission abatement burden of the production side of each sector.

The model uses economy, energy, and GHG related datasets of Korea, such as the social accounting matrix from the input-output table [24], energy balance data [25], and GHG emissions data [26], setting 2015 as the base year. Table 3 shows the descriptive statistics for the important variables for each industry. The production sector comprises 18 sectors, which consist of four energy sectors (with energy commodities divided into coal, gas, oil, and electricity) and 14 non-energy sectors.

### 3.2. Modeling the Electricity Sector

**Figure 2:**
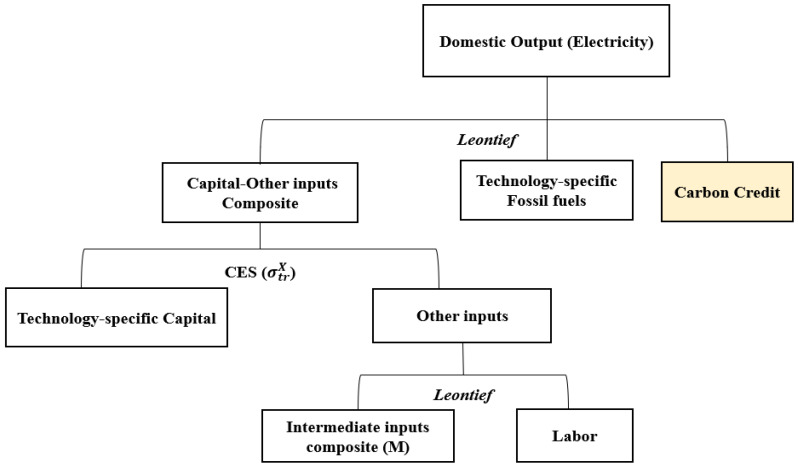
Nested production structure of the electricity sector.

The production structure of the electricity sector is different from other industries, as presented in Figure 2. Electricity is generated through various technologies using various resources such as coal, gas, oil, nuclear, hydro, solar PV, wind, and other renewables. Each technology shares the same production structure described in Figure 2 and produces the same good (electricity). Technologies which do not rely on fossil fuels as input (such as nuclear, hydro and renewable energies) have zero values for the ‘technology-specific fossil fuels’ input in Figure 2. In this model, capital input for each generation technology is treated as the fixed factor belonging to each technology. This fixed factor reflects the capacity restrictions of each generation technology [17,27] and is integrated with other inputs consisting of intermediate composites and labor inputs to produce electricity. The substitution elasticities of $\sigma_{tr}^{X}$ within the CES production structure are calibrated consistently with exogenously given supply elasticities of each generation technology [17,27,28]. The exogenously given supply elasticities are used to calibrate the substitution elasticities of CES functions for generation technologies in the model, and the detailed procedures are explained in Rutherford [28].

Supply elasticity represents how flexibly each technology can change its generation capacity in response to demand. The model sets the lower values of supply elasticities for generation technologies and sources constructed under the government plan over the long term that supply baseload electricity, such as coal power plants and nuclear power plants. The model sets relatively higher values for those that could be built in short periods and operated flexibly, such as gas power plants and renewable energy power plants.

This study divides the power generation sector into detailed generation technologies and sources, following previous studies [15,16,17,18,19,20,21]. The structure of inputs for each generation technology and source are elaborated, which makes it possible to see the effect of support policies for specific renewable energy technology. However, the power generation sector in Korea’s official input-output table by the Bank of Korea [24] is divided only into categories such as thermal, hydro, nuclear, and renewable (as well as self-generation and miscellaneous). Various renewable energy technologies and sources, such as solar PV, wind, and bioenergy, are all combined into a single renewable energy category, making it challenging to observe the effects of expanding a specific renewable energy generation technology. Thus, to overcome such difficulties, this study employs the input-output table for renewable energy in Korea, which was recently built by Kim and Suh [14]. Kim and Suh [14] gathered data on production costs for each renewable energy technology and source by surveying companies in related industries and divided the renewable energy sector in the input-output table into detailed categories, including solar PV, wind, and other renewables.

Table 4 shows the intermediate input structure in accordance with the sub-classification of the renewable energy source by Kim and Suh [14]. Regarding solar PV generation, the input portions of the CHE and ECT industries, which produce polysilicon solar panels, inverters, and power generation systems, are large. Regarding wind power generation, the input portions of the MAC and ECT industries, which produce wind turbines, inverters, and generation systems, are large.

Meanwhile, the category of “thermal” power in the input-output table was divided into coal, gas, and oil power generation sectors, given the power generation and the fuel consumption volumes. Thus, the power generation sector in the model was divided into coal, gas, oil, nuclear, hydro, solar PV, wind, and other renewable energy sectors. Here, the capital allocated to each power generation technology and source acts as fixed capital for each power generation technology (Figure 2).

### 3.3. Scenario Settings

The scenarios used for analysis in this study are described in Table 5. First, scenarios were set up such that the shares of solar PV and wind power generations increased to about 7% of the total power generation (labeled as SOL and WIN scenario, respectively, in Table 5). Here, 7% was set based on the solar PV and wind power generation targets in Korea’s “Renewable Energy 3020” plan [4,5,6]. Meanwhile, the increase in renewable energy sources is achieved through financial support from the government. Currently, renewable energy expansion policies in Korea (such as the renewable portfolio standard (RPS) and FIT) are designed to cover the required financing needs via taxation in the power generation sector [29]. Studies such as Böhringer et al. [15,18] have highlighted that policy effects on the economy vary significantly depending on the funding source for renewable energy expansion. Therefore, scenarios were added, which compare the case of financing the renewable expansion via a lump sum tax (LT in Table 5) to that of financing via taxation in the electricity sector (ET in Table 5) and that of financing via levying a carbon tax on fossil fuel usage and related GHG emission (CT in Table 5). The rate of each tax is determined in the model to hit the specific renewable expansion policy targets.

Additionally, Korea introduced the emissions trading scheme (ETS) in 2015. Emission regulations, such as the ETS, increase the production costs of fossil fuel power generation, thus resulting in the increased competitiveness of renewable energy generation. Given these factors, scenarios were added to compare the impacts on the economy when the shares of renewable energy generation increase under the ETS, through which 20% of total emissions are reduced (ETS in Table 5).

In actual, the Korean ETS covers around 70% of the entire GHG emission from the economy. Manufacturing industries and power sector, buildings, waste sector, and domestic aviation sector are included, and emissions from households and automobiles are excluded in the ETS. Most of the carbon credits are freely allocated (around 97% in 2020). The Korean ETS has its own specific design characteristics related to flexibilities (such as banking and borrowing, offsets, and market stability provisions) and compliances (monitoring, reporting, verifying, and enforcement) [30,31,32,33].

However, in this model, the ETS is very simply simulated to conceptually represent the regulation on GHG emissions. In the model, the entire economy is under the scope of the ETS and 100% of carbon credits are auctioned. The government initially owns the carbon credits. When there is no cap on GHG emissions (ETS not implemented), the government has inexhaustible amount of carbon credits and the price of carbon credit becomes zero. However, when the ETS is implemented, the total cap is set at 80% of the base year emission, which forces emission reduction by 20% compared to the base year emission, for the entire economy. In the model, as described in Figure 1; Figure 2, using fossil fuels as inputs requires purchasing of carbon credits proportional to the amount of GHG emissions from the fuels. The price of carbon credit is then determined in the model by the interaction of supply and demand. Use of fossil fuels generates demand for the carbon credits and the supply of carbon credits is determined by the level of the cap in the ETS.

## 4. Analysis Results

This section first describes the results of simulating the scenario wherein renewable energy is expanded when there is no regulation on GHG emissions. Subsequently, the results of simulating the scenario wherein renewable energy is expanded, and GHG emissions are regulated through the ETS, are explained.

### 4.1. Effects of Solar PV and Wind Generation Expansion

Table 6 shows the results of the scenarios wherein the share of solar PV power generation increases to about 7%, following the renewable energy expansion policy. It contains the results of one scenario where financing is provided through a lump-sum tax (SOL-LT) and another scenario where financing is provided through taxation in the electricity sector (SOL-ET) and the other scenario where the financing is provided through an economy-wide carbon tax (SOL-CT). When the share of solar PV power generation increases from 0.7% in the base year to about 7.0% due to government subsidies, the changes in welfare (real consumption of goods by the representative household), GDP, wage rate, electricity price, and GHG emissions are observed.

When the share of solar PV power generation increases to about 7%, welfare decreases by 0.84%, 0.85% and 0.93% for SOL-LT, SOL-ET, and SOL-CT scenarios, respectively. Additionally, GDP declines by 0.37%, 0.44%, and 0.52% for SOL-LT, SOL-ET, and SOL-CT scenarios, respectively. The decrease seems to originate from both the government and the private sectors, which reduce consumption due to the burden of financing government subsidies for the solar PV power expansion. The impact of solar PV expansion on the demand for labor can be seen through changes in the wage rate (the price of labor). Since the model assumes fully-mobile labor with full employment, the increase in the wage rate can be interpreted as an increase in the demand for labor (which can be interpreted as an increase in employment in a real economic situation). The demand for labor, which can be viewed regarding changes in wage rate, shows different results in SOL-LT, SOL-ET, and SOL-CT. It increases by about 0.37% in SOL-LT and decreases by about 0.19% and 0.37% in SOL-ET and SOL-CT. However, the electricity price drops by 7.08% in SOL-LT and increases by 3.90% in SOL-ET. In SOL-CT, the electricity price shows a slight decrease of 1.63%. In SOL-LT, the amount of solar PV power generation increases due to the renewable energy subsidies, which results in an overall increase in the total power generation. However, the demand for electricity reduces due to the decrease in GDP. Thus, electricity prices fall significantly. The demand for labor increases mainly due to the increase in solar PV power generation. That is, the demand for labor increases as the existing fossil fuel generation sector, which is capital- and energy-intensive, is replaced by the solar PV power sector, which is (relatively) labor-intensive and requires more intermediate goods. However, in SOL-ET, the price of electricity increases as solar PV power generation increases since the subsidy for renewable energy originates from taxation in the power generation sector. The tax in the power generation sector appears to be a burden on the existing fossil fuel power generation sector, which reduces its demand for labor and results in a decrease in the overall demand for labor. In SOL-CT, the decrease in GDP and welfare is larger than other scenarios, since the carbon tax induces additional costs on energy-intensive industries as well as electricity sector, which comprise a large part of the Korean economy. The decreased demand for labor and electricity due to the reduced economic activity is causing the wage rate and electricity price to decrease. Meanwhile, renewable energy expansion replaces existing coal and gas power generation, thereby reducing GHG emissions. GHG emissions decrease by 6.42% in SOL-LT, 9.23% in SOL-ET, and 14.90% in SOL-CT when the share of solar PV generation reaches 7%. The decline in SOL-CT is largest because the carbon tax reduces production activity of energy intensive industries (such as the basic metal industry) significantly.

Table 7 shows the changes in welfare, GDP, wage rate, electricity prices, and GHG emissions when wind power generation increases from 0.2% to about 7% due to government subsidies. Overall, it shows a similar trend in the increase in solar PV power; however, the size of the effect is slightly larger. For example, as wind power generation increases to 7%, GDP shows a decline of about 0.63% in WIN-LT, showing a larger decline relative to SOL-LT (0.37%). This difference seems to be caused by the initially lower share of wind power generation (about 0.2%), as well as the differences in the cost structure of solar PV and wind power generation. Alsharif et al. [34] pointed out that large seasonal variation of wind speed and direction, the long distance between metropolitan areas and potential wind farms, and the lack of domestic manufacturers with competitiveness as factors slowing the diffusion of wind generation (compared to solar PV) in Korea. The results in Table 7 also seems to partly reflect these factors. WIN-ET shows a slightly greater decrease in GDP compared to WIN-LT, which seems to be due to the slowdown in industrial activity as the price of electricity increases significantly. However, the largest decrease in welfare and GDP is shown in the WIN-CT scenario. In WIN-LT, electricity prices drop sharply due to the combination of a decrease in electricity demand, resulting from the slowdown in industrial activities and the subsidy effect in the renewable energy sector. However, in WIN-ET, electricity prices rise sharply by 8% as the cost of wind power expansion is imposed on the power generation sector. When the carbon tax is imposed (as in WIN-CT), there are two kinds of actions which affect the electricity price in opposite directions. One is the decreased demand for electricity due to the reduced economic activity, and the other is the increased cost of power generation due to the carbon tax imposed on the fossil fuel generation sources. In SOL-CT, the interaction between the two effects resulted in decreasing the electricity price. However, in WIN-CT, the effects seem to operate in a way to increase the electricity price.

Korea’s GHG emissions reduce due to the decrease in GDP and the decarbonization of the power generation sector. In the WIN-ET scenario, such factors are also combined with increased electricity prices and reduced power generation, resulting in a decrease in GHG emissions by 10.60%. Additionally, in WIN-CT, the emission decreases by 18.20% compared to the base year situation, due to decreased activities in the energy intensive industrial sectors.

Table 8 and Table 9 show the effect of expanding the share of solar PV and wind power generation on each industry. While most industries show a slight decrease in output due to decreased GDP and consumption, the upstream industry, which supplies parts and modules to the solar PV and wind power generation sectors, shows signs of stimulation. When the share of solar PV power generation increases, the industry with the largest increase in output among the manufacturing industries is the CHE industry, which provides components for PV generators such as polysilicon solar panels. In SOL-LT, the output of the CHE industry increases by 3.65% as the share of solar PV increases to 7%. The output of the MAC and the ECT industries, which provide materials used in solar PV panels, also increases. When the share of wind power generation increases, the increase in output is most prominent in the MAC industry. In WIN-LT, the output of the MAC industry increases by 4.90%. The MAC industry supplies major modules, such as turbines, for wind power generators. The output of the ECT industry, which supplies modules such as inverters, also increases by 1.30%. Meanwhile, the output of the AUT industry decreases due to the increased price of intermediate goods required (e.g., metal products, and mechanical and electronic equipment) by the expansion of solar PV and wind power facilities. Moreover, since automobiles and transport equipment are mainly final consumer goods, the reduction in consumption due to the tax burden seems to have influenced the reduction in output of the automobiles and transport equipment industry. In the IRO industry, the output increases in SOL-LT and WIN-LT, while the output decreases in SOL-ET, SOL-CT, WIN-ET, and WIN-CT. The decrease is far more pronounced in the carbon tax (CT) scenarios. IRO is one of the industries that consumes large amounts of electricity and coal. In SOL-LT and WIN-LT, the production costs of the IRO industry decrease due to the decreased electricity prices; thus, the output increases. In SOL-ET and WIN-ET, the opposite effects are observed, caused by increased electricity prices. In SOL-CT and WIN-CT, the decrease in IRO industry is very large because the industry requires large amounts of coal as a source of energy as well as raw material. A similar trend is observed in the NMP industry, which also consumes large amounts of electricity and fossil fuels.

Table 10 and Table 11 show the changes in the amount of total power generation and coal and gas generation when solar PV and wind power generation are expanded. The total power generation of the power industry increases by 2.63% and 2.81%, respectively, in the SOL-LT and WIN-LT scenarios, due to the support for the renewable energy sector. However, in the SOL-ET and WIN-ET scenarios, the amount of power generation decreases by 2.92% and 4.81%, respectively, due to the tax burden on the power generation industry and the increased electricity prices. In the SOL-CT and WIN-CT scenarios, the total power generation decreases by 0.65% and 1.80%, respectively. Across all the scenarios, the amount of gas generation decrease is significantly larger than the decrease in the coal generation. Renewable energy sources mainly replace gas power generation because gas generation plants can operate flexibly, and the fuel cost is higher relative to coal generation plants. However, when the carbon tax is applied (SOL-CT and WIN-CT), coal power generation amount also shows a substantial decrease (−23.63% for SOL-CT and −28.48% for WIN-CT) due to its high GHG intensity.

### 4.2. Effects of Solar PV and Wind Power Expansion in the Presence of Emission Regulations

This section observes the effects of expanding solar PV and wind power generation when GHG emission is regulated to achieve a 20% reduction through the ETS on the entire economy. That is, it observes the interaction between emission regulations and renewable energy expansion policies.

When the economy-wide ETS is implemented, the prices of existing fossil fuel power generation sources rise, thereby increasing the competitiveness of renewable energy. Therefore, even if no separate support policy exists, the share of solar PV and wind power generation expands to about 1.0% and 0.3%, respectively. Moreover, the price of carbon credit, which allows for the emission of 1 ton of GHG (TCO_2_eq), reaches about 42.57 thousand KRW.

Table 12 shows the macroeconomic effects of increasing solar PV power generation when emissions are regulated (when GHG emission is regulated to achieve a 20% reduction through the ETS on the entire economy). Even if there is no government support policy due to emission regulations, the share of solar PV power generation increases to about 1.0% due to the implementation of the ETS.

In the case of SOL-LT/ETS, GDP decreases as the share of solar PV power generation increases to about 7%. However, the magnitude of the decrease is not large relative to the SOL-LT scenario. In SOL-LT/ETS, GDP decreases by about 0.13%p, from −0.54% to −0.67%, which is far smaller than the 0.37% decrease in the SOL-LT scenario. When emission regulations are implemented, the demand for production factors, such as labor and capital decreases as industrial activity, slows, and the cost of electricity generation from fossil fuels rises due to payment for carbon credits. Thus, renewable energy expansion policy has the positive effect of revitalizing the production activities of related industries, increasing the demand for labor and capital, and alleviating electricity price increases. Hence, the decrease in GDP due to renewable energy expansion is relatively smaller. It can be said that such results support the rationality of stimulus packages, such as the “Green New Deal,” as argued by Fulton et al. [35], Böhringer et al. [17], and Oh et al. [8]. During the economic downturn, the renewable energy expansion policy can facilitate the usage of surplus capital and production capacity and address unemployment. A similar trend can be found when seen from changes in welfare. The decrease in welfare in SOL-LT/ETS through the expansion of solar PV is about 0.55%p, from −0.51% to −1.06%, which is smaller than 0.84% decrease in the SOL-LT scenario.

In the SOL-LT/ETS scenario, the demand for labor, which can be viewed in terms of changes in wage rates, initially shows a decrease of about 1.95% due to the decrease in industrial activity when the ETS is implemented. However, when the share of solar PV power is expanded to 7%, the demand for labor decreases by 0.96%. Most of the increase in the demand for labor comes from the expansion of the solar PV industry. In SOL-LT/ETS, the electricity price increases rapidly by 12.97%, as fossil fuel prices rise due to the ETS. However, as the government begins financing the solar PV power expansion, the electricity prices decrease significantly to 2.81%. The price of carbon credit for the 20% GHG reduction reduces from 42.57 thousand KRW to 27.42 thousand KRW, as the share of solar PV power generation expands. Given the decarbonization of the power sector, the amount of carbon emissions decreases, reducing the demand as well as the price of carbon credit.

Meanwhile, in SOL-ET/ETS, when the share of solar PV power is expanded to 7%, the extent to which the demand for labor and electricity prices increases (from −1.95% to −1.22%) and decreases (from 12.97% to 11.12%), respectively, is smaller relative to the case of SOL-LT/ETS. In SOL-ET/ETS, the demand for labor in the existing fossil fuel-based power generation industry is reduced due to the taxation in the power generation industry to finance renewable energy expansion. Many factors influence the electricity price: while the taxation may cause electricity prices to rise, the decarbonization of the power generation industry, which results in decreased demand for carbon credits from the power sector, as well as the reduced demand for electricity due to a slowdown in the overall activity level of the economy, may cause electricity prices to fall. Given a combination of these effects, the increase in the electricity price falls from 12.97% to 11.12%, as the share of solar PV power increases. However, the price of carbon credit reaches 22.8 thousand KRW in the case of SOL-ET/ETS, which is lower than that of SOL-LT/ETS as the share of solar PV power reaches 7%. The reduction in fossil fuel generation as a result of the tax on the entire power generation industry seemingly explains the result. Accordingly, the demand for carbon credit is also reduced compared to that of SOL-LT/ETS. A slowdown in the overall production activity of the economy may also contribute to the decline in the price of carbon credits.

Table 13 shows the macroeconomic effects of increasing wind power generation when emissions are regulated (when GHG emission is regulated to achieve a 20% reduction through the ETS on the entire economy). Even without a government support policy due to emission regulations, the share of wind power generation will increase to about 0.3%.

In WIN-LT/ETS, when the share of wind power generation increases to about 7% with subsidies, GDP decreases from −0.54% to −0.90%. That is, there is an additional drop in GDP of approximately 0.36%p, which is less than the 0.63% reduction in the WIN-LT scenario. Although the extent of the reduction varies, the overall trend is similar to the case of solar PV expansion. Meanwhile, in WIN-ET/ETS, electricity prices rise until the share of wind power generation increases to 5% and then fall. This change in the direction for electricity prices seems to result from the interaction between the tax effect on the power generation industry (which causes electricity prices to rise), and the decarbonization of the power generation industry as well as the reduced demand for electricity (which cause electricity prices to fall).

In Table 14 and Table 15, the effect on each industry is observed under the SOL-LT/ETS, SOL-ET/ETS, WIN-LT/ETS, and WIN-ET/ETS scenarios, where the share of solar PV and wind power generation increases to 7% under the ETS. The industry most negatively affected by the emission regulations is the IRO industry, which consumes large amounts of coal. The output of the IRO industry decreases by about 16.25% when the ETS is implemented. However, the level of output decrease in the IRO industry is alleviated to about a decrease of 9%–11% when the share of renewable energy generation reaches 7%. This output increase in the IRO industry results from an increase in demand for intermediate materials required to produce renewable energy generators and a decrease in the demand and price of carbon credit due to the decarbonization of the power generation industry. The TRN industry, which is affected by the rise in oil prices due to the ETS, shows a similar trend. As the share of renewable energy increases, the output of the TRN industry increases due to the decrease in the price of carbon credit. However, the increase in the share of renewable energy causes an increase in output in the upstream industry. Regarding the CHE industry, the output shows a 2.90% decrease due to emission regulations. However, as the share of solar PV generation increases in the SOL-LT/ETS scenario, the changes in the output of the CHE industry become positive (1.49% increase) due to the increase in the demand for intermediate materials (e.g., polysilicon for solar PV panels). Similarly, in the MAC industry, the output is reduced by 1.45% due to emission regulations. However, in the WIN-LT/ETS scenario, the output of the MAC industry increases by 3.52% when the share of wind power generation expands to 7%.

Meanwhile, some industries benefit when there are emission regulations, such as industries wherein the share of energy-related costs in total production costs is small, as well as export-oriented industries that indirectly benefit from lower capital and labor costs due to the slowdown in overall industrial production. The ECT industry is a typical case. The output of the ECT industry increases by 4.38% when the ETS is implemented. However, the increase in the output of the ECT industry decreases as solar PV and wind power generation increases. This situation arises because the increasing share of renewable energy generation increases the demand for labor and capital, which weakens the relative cost competitiveness enjoyed by the ECT industry.

Table 16 and Table 17 show the changes in coal and gas generation when solar PV and wind power generation are expanded, given emission regulations. In the absence of subsidies for solar PV or wind power expansion, GHG emission regulations significantly reduce coal generation with large emission intensity and increase gas generation with relatively low emission intensity. A drastic change in the electricity generation mix, with coal power generation decreasing by 33.43% and gas generation increasing by 18.90%, occurs. Thus, the total power generation decreases by 6.63%. Meanwhile, the level of decrease in total power generation becomes smaller with the expansion of renewable energy in the SOL-LT/ETS and WIN-LT/ETS scenarios. This situation arises because the existing fossil fuel power generation sector indirectly benefits from the reduced price of carbon credit induced by renewable energy expansion. However, no remarkable change is observed in the total power generation with the expansion of renewable energy in the SOL-ET/ETS and WIN-ET/ETS scenarios. This situation seems to arise because the financing for renewable energy expansion comes from the power generation industry.

When renewable energy expands, the amount of gas power generation, rather than coal generation, is reduced. In all scenarios, the expansion of renewable energy generation rapidly replaces gas power generation. Gas power generation initially increases by 18.90% when the ETS is implemented. However, it shows a significant drop to 26–40% relative to the base year, as the share of renewable energy generation increased to 7%. Moreover, coal power generation shows a slight increase when renewable energy expands under ETS.

Böhringer and Rosendahl [36] also observed the phenomenon wherein sectors, such as coal power generation, with large emission intensity benefit from renewable energy expansion when there are overlapping regulations, such as the ETS and the renewable energy expansion policy. According to Böhringer and Rosendahl [36], when the share of renewable energy increases, the amount of power generated by fossil fuels should decrease (Effect 1). Moreover, GHG emissions reduce accordingly, and the price of the carbon credit reduces (Effect 2). Effect 1 burdens all fossil fuel sources to decrease their outputs. However, Effect 2 offers incentives to power sources and industries with high emission intensity (e.g., coal power generation and the IRO industry). Thus, power sources with high emission intensity, such as coal generation, indirectly benefit from the interaction between Effect 1 and Effect 2. These effects are also well observed in the simulation results of this model. When the renewable expansion policies are adopted, the amount of coal and gas power generation both decreases as seen in Table 10 and Table 11 (Effect 1). However, when the overlapping regulations, such as ETS and renewable energy expansion policies, exist in the power generation sector, renewable energy expansion can provide incentives for industries and power sources with higher emission intensity (Effect 2). In the scenarios where the overlapping generation exists (SOL-LT/ETS, SOL-ET/ETS, WIN-LT/ETS, WIN-ET/ETS), the expansion of renewable energy works in a way to decrease the price of carbon credit (Table 12 and Table 13), resulting in increase or maintenance of the amount of coal power generation compared to the gas power generation (Table 16 and Table 17). For example, in the SOL-LT/ETS scenario, the amount of coal power generation increase from −33.43% to −31.25% while the amount of gas power generation sharply decreases from 18.90% to −26.68%, as the share of solar PV increases. The IRO industry, which has the highest GHG intensity, also follows the similar pattern under the overlapping regulation. However, the situation, where the expansion of renewable energy replaces gas power generation instead of coal power generation, is better to be avoided. This is because not only does it cause GHG emission problems, but it also increases the emissions of air pollutants, such as PM_2.5_, making air pollution and public health problems in Korea more serious [11].

The EU ETS and other schemes are adopting measures to avoid the phenomenon, where the overlapping regulations incentivize the use of fossil fuels, and they are mostly concerned in preventing the carbon price from falling too much. The measures include adjusting the reduction targets, limiting the use of international carbon credits, and using the market stability reserve to either injects or withdraws liquidity into the market to stabilize prices [37]. Adjusting the coverage of ETS sectors, decreasing the amount of grandfathered allowances to the renewable energy sector, and interaction between Renewable Energy Certificates and carbon markets are also suggested [38]. The Korean ETS is equipped with various design options to maintain the price level of carbon, such as use of the market stability reserve, regulating banking and borrowing of credits, and restricting the use of the international offsets. These measures can be applied when the overlapping regulation becomes problematic in practice.

## 5. Summary and Future Research Directions

This study examines the effects of increased renewable energy power generation on the Korean economy and its industrial sectors using the CGE model, which divides the power generation sector into detailed generation technologies and sources. It includes scenarios that assume that the shares of solar PV and wind power generation respectively expand to approximately 7%. Scenarios are added to observe the effects of renewable energy expansion when GHG emissions are regulated. Moreover, scenarios compared the case when financing for renewable energy expansion was made through a lump-sum tax with the case when the financing was made via taxation on the power generation industry and the case when the financing was made via levying a carbon tax on fossil fuel usage.

The main implications of the paper are as follows: First, solar PV and wind power generation expansion through government subsidies have a negative effect on GDP. The negative effect was simulated to be larger in the case of wind expansion than in the case of solar PV expansion. Second, the solar PV and wind power expansions lead to an output increase in the upstream industries, such as the CHE and ECT industries, in the case of solar PV expansion, and the MAC and ECT industries, in the case of wind power expansion. Third, if the renewable energy expansion is financed with a lump-sum tax, electricity prices decline, and the demand for labor increases. However, if the renewable energy expansion is financed by taxation in the power generation industry, the opposite effects can be observed. Levying a carbon tax to finance renewable expansion is simulated to be most effective in terms of GHG emission reduction, however, the negative impact on the economy is also largest among scenarios. Fourth, the increased share of renewable energy sources mostly replaces gas generation.

However, when there are emission regulations, such as the ETS, simulating the renewable energy expansion policy reveals the following: First, the extent of the GDP decline that results from solar PV and wind power generation expansion reduces, as compared to the scenarios without emission regulations. Second, when overlapping regulations, such as ETS and renewable energy expansion policies, exist in the power generation sector, renewable energy expansion can provide incentives for industries and power sources with high emission intensity.

Korea is implementing GHG emission regulations, such as the ETS. Such regulations are expected to be strengthened in the future. Although emission regulations can be burdensome on the economy, resulting in decreased demand for labor and capital, the results show that green stimulus policies, such as renewable energy expansion, can help achieve decarbonization of the economy, develop related industries, and maintain or increase employment. However, if the financing for renewable energy expansion is obtained only from the power generation sector, the significant slowdown in economic activity due to increased electricity prices and the reduction in the demand for labor in the existing fossil fuel generation sector may offset the increase in employment due to renewable energy expansion and adversely affect employment. Hence, it seems necessary to diversify the financing for renewable energy expansion. Furthermore, Korea’s power generation industry operates under the overlapping regulations of the ETS and the renewable energy expansion policy. It would be necessary to adjust policies such that the overlapping regulations do not act as incentives for electricity sources with high emission intensity, such as coal power generation.

This is the first study on Korea that utilizes a realistic input structure of major renewable energy sources using the input-output table. However, it has limitations and must be supplemented with further studies. The limitations and future research directions include the following: First, the simulation of the labor market is very simple; the effect on the employment perspective is observed through changes in the unit price of labor. This is due to the lack of information on the number of employees in the specific renewable industries. Nonetheless, more realistic results can be achieved by introducing choices between labor and leisure, restrictions on free mobility of labor, and changes in the endowment of labor. The sophisticated modeling of the labor market will produce various policy implications related to the renewable energy policies, such as the impact of the ‘Green New Deal’ on job creation, observing the effects of vocational training in renewable energy sector, observing the direct and indirect impacts of renewable policies on unemployment, and others. Second, our analysis is conducted with a static model, so we could not incorporate potentially important dynamic elements of the policies. Instead we focus on long-run impacts when the renewable policies are fully built-out. The dynamic CGE model requires a reliable Business-As-Usual (BAU) scenario for the future, where the additional renewable policies are not implemented yet. Due to the characteristics of this model, the BAU scenario should have various projections on macro variables, sectoral energy demand, sectoral GHG emission, fuel mix for electricity generation, renewable energy share, and cost of renewable generation technologies. However, it was not easy to find appropriate projections for these variables. Projections from various studies were based on different assumptions for different periods, and most of them were already incorporating the renewable expansion policies (which was not suitable for generating the BAU scenario). Efforts are needed for future studies to generate a good and reliable BAU scenario with suitable projections, and to build and operate the dynamic CGE models. Third, another important dynamic missing from the static model is technical change. The cost of renewable energy is expected to decrease significantly in the future. However, studies forecasting the cost for each renewable technology are still scarce in Korea. Zhou and Gu [39] used the learning curve analysis for wind and solar PV power generations for US. If such a study is available for Korea, the cost projection can be incorporated in the dynamic CGE model to forecast the results of policies accurately.

## 6. Conclusions

The results show that renewable expansion policies have negative effects on GDP. However, the magnitude of the GDP decline becomes smaller when GHG emissions are regulated. The expansion of renewable energy induces the growth of upstream industries which supply components for renewable generation modules. Regarding employment, the renewable expansion policy can increase the demand for labor. However, the direction and the extent of the effect vary depending on the funding source. When overlapping regulations, such as the emission trading scheme and renewable energy expansion policies, exist in the power generation sector, the renewable energy expansion policy could provide incentives for GHG emission-intensive power sources.

## Figures and Tables

**Table 1:** Impacts on key outcomes of renewable expansion policies.

Authors	Welfare Impacts	GDP Impacts	Employment Impacts	GHG Emissions
Böhringer and Löschel [16]	Negative	nr	nr	Decrease
Böhringer et al. [17]	Negative	nr	Negative	nr
Böhringer et al. [15]	Positive/Negative	nr	Positive/Negative	nr
Qi et al. [19]	nr	nr	nr	Decrease
Dai et al. [20]	Negative	Negative	Negative	Decrease
Mu et al. [21]	nr	Negative	Positive/Negative	nr

Note: nr = not reported.

**Table 2:** Key parameters in the model.

Parameter	Description	Value	Comments
$\sigma_{VA}$	Elasticity of substitution between labor and capital	0.02~0.46	Varies by sector
$\sigma_{VAE}$	Elasticity of substitution between energy and value-added	0.00~0.60	Varies by sector
$\sigma_{M}$	Elasticity of substitution among intermediate inputs	0.00~0.60	Varies by sector
$\sigma_{VAEM}$	Elasticity of substitution between intermediates and the aggregate of value-added and energy inputs	0.00~1.17	Varies by sector
$\sigma_{OG}$	Elasticity of substitution between oil and gas energy inputs	0.5	All sectors
$\sigma_{OGC}$	Elasticity of substitution between the aggregate of oil and gas and coal energy inputs	0.5	All sectors
$\sigma_{ELE}$	Elasticity of substitution among different energy inputs (coal, gas, oil, and electricity)	0.5	All sectors
$\sigma_{DM}$	Elasticity of substitution between domestic goods and imports	4	All sectors

**Table 3:** Industry classification used in the model and descriptive statistics for each industry.

Industry Description		Industry Label	Outputs (Unit: 1 Trillion KRW ^a^)	Electricity Intensity ^b^ (Unit: %)	GHG Emission (Unit: Million Ton CO2eq.)
Energy sectors	Coal	COA	0.8	4.5	0.0
	Oil	OIL	109.6	1.6	5.9
	Gas	GAS	28.6	0.1	0.0
	Electricity and heat	ELE	64.2	2.4	242.6
Non-energy sectors	Agriculture, forestry, and fishing	AFF	61.4	1.2	24.7
	Mining and quarrying	MIN	3.9	2.2	0.2
	Food and tobacco	FOO	122.1	0.8	1.8
	Textile and leather	CLO	82.7	1.3	1.5
	Paper, pulp, and print	PPP	42.9	3.1	0.7
	Chemical and plastic products	CHE	242.0	2.2	38.8
	Nonmetallic minerals products	NMP	39.7	3.2	46.8
	Basic metal products	IRO	125.3	4.7	108.3
	Fabricated metal products, machinery, and equipment	MAC	221.1	1.1	1.9
	Electric, electronic, and precision products	ECT	381.7	1.1	11.8
	Automobiles and transport equipment	AUT	258.0	0.6	1.0
	Construction	CON	223.6	0.3	7.1
	Transportation	TRN	138.6	0.9	61.1
	Commercial and public services	SER	1687.4	1.4	52.5

Note: ^a^ 1 US dollar = 1232 Korean Won (KRW) as of 23 April 2020. ^b^ Electricity Intensity is calculated based on the cost-share of electricity in output.

**Table 4:** Intermediate input structure by renewable energy technology and source (unit: %).

Industries	Solar PV	Wind	Other Renewables
COA	0.00	0.00	0.00
OIL	0.51	0.45	4.41
GAS	0.23	0.20	1.97
ELE	0.70	0.61	6.08
AFF	0.04	0.03	0.03
MIN	0.00	0.00	0.00
FOO	0.41	0.29	0.26
CLO	1.42	1.01	0.91
PPP	0.15	0.12	6.72
CHE	31.54	7.69	32.92
NMP	0.02	0.02	0.01
IRO	0.00	0.00	0.00
MAC	13.20	47.94	5.07
ECT	25.00	25.01	9.69
AUT	0.80	0.46	0.82
CON	0.26	0.23	0.35
TRN	0.71	0.41	0.73
SER	25.02	15.54	30.05
Total	100.0	100.0	100.0

**Table 5:** Scenarios used in the analysis.

Scenario Label	Description
SOL-LT, SOL-ET, SOL-CT	The scenario that increases the share of solar PV power generation to about 7% via financial support. SOL-LT: financing subsidy through lump-sum tax SOL-ET: financing subsidy through taxation in the electricity sector SOL-CT: financing subsidy through a carbon tax on fossil fuel usage
WIN-LT, WIN-ET, WIN-CT	The scenario that increases the share of wind power generation to about 7% via financial support. WIN-LT: financing subsidy through lump-sum tax WIN-ET: financing subsidy through taxation in the electricity sector WIN-CT: financing subsidy through a carbon tax on fossil fuel usage
SOL-LT/ETS, SOL-ET/ETS, WIN-LT/ETS, WIN-ET/ETS	Each scenario is under the economy-wide ETS, through which 20% of total emissions are reduced

**Table 6:** Macroeconomic Effects of solar PV generation expansion (% changes from the base year).

Scenarios	Indexes	Share of Solar PV Generation (%)			
		0.7 ^a^	3.0	5.0	7.0
SOL-LT	Welfare	0.00	−0.21	−0.50	−0.84
	GDP	0.00	−0.09	−0.22	−0.37
	Wage rate	0.00	0.10	0.21	0.37
	Electricity price	0.00	−1.89	−4.13	−7.08
	GHG emission	0.00	−2.20	−4.27	−6.42
SOL-ET	Welfare	0.00	−0.22	−0.51	−0.85
	GDP	0.00	−0.12	−0.27	−0.44
	Wage rate	0.00	−0.09	−0.16	−0.19
	Electricity price	0.00	1.84	3.24	3.90
	GHG emission	0.00	−3.07	−6.06	−9.23
SOL-CT	Welfare	0.00	−0.24	−0.56	−0.93
	GDP	0.00	−0.13	−0.31	−0.52
	Wage rate	0.00	−0.13	−0.25	−0.37
	Electricity price	0.00	−0.39	−0.85	−1.63
	GHG emission	0.00	−5.30	−10.10	−14.90

Note: ^a^ This column indicates base year situation without renewable expansion policy.

**Table 7:** Macroeconomic effects of wind generation expansion (% changes from the base year).

Scenarios	Indexes	Share of Wind Generation (%)			
		0.2 ^a^	3.0	5.0	7.0
WIN-LT	Welfare	0.00	−0.44	−0.87	−1.37
	GDP	0.00	−0.20	−0.40	−0.63
	Wage rate	0.00	0.10	0.20	0.34
	Electricity price	0.00	−2.31	−4.56	−7.52
	GHG emission	0.00	−2.60	−4.57	−6.64
WIN-ET	Welfare	0.00	−0.45	−0.88	−1.36
	GDP	0.00	−0.24	−0.47	−0.71
	Wage rate	0.00	−0.20	−0.34	−0.44
	Electricity price	0.00	3.82	6.34	7.97
	GHG emission	0.00	−4.05	−7.23	−10.60
WIN-CT	Welfare	0.00	−0.48	−0.97	−1.50
	GDP	0.00	−0.27	−0.54	−0.84
	Wage rate	0.00	−0.27	−0.49	−0.72
	Electricity price	0.00	0.21	0.39	0.30
	GHG emission	0.00	−7.50	−13.00	−18.20

Note: ^a^ This column indicates base year situation without renewable expansion policy.

**Table 8:** Changes in output by industry according to the increase in the share of solar PV power generation (% changes in output from the base year).

Scenarios		SOL-LT			SOL-ET			SOL-CT		
Share of Solar PV Generation (%)		3.0	5.0	7.0	3.0	5.0	7.0	3.0	5.0	7.0
Industries	AFF	−0.17	−0.39	−0.65	−0.13	−0.31	−0.52	−0.10	−0.26	−0.44
	PPP	0.02	0.04	0.07	−0.13	−0.25	−0.35	−0.01	−0.03	−0.04
	MIN	−0.05	−0.04	0.03	−0.23	−0.38	−0.46	−0.32	−0.61	−0.88
	FOO	−0.15	−0.35	−0.59	−0.14	−0.32	−0.53	−0.10	−0.26	−0.44
	CLO	−0.09	−0.20	−0.31	−0.18	−0.37	−0.55	0.24	0.46	0.68
	CON	0.00	0.00	0.00	0.00	−0.01	−0.01	0.00	−0.01	−0.01
	TRN	−0.09	−0.19	−0.31	−0.07	−0.15	−0.24	−0.47	−1.00	−1.60
	SER	−0.05	−0.13	−0.22	−0.06	−0.14	−0.23	−0.04	−0.12	−0.20
	MAC	0.16	0.36	0.60	0.07	0.17	0.31	−0.04	−0.06	−0.07
	ECT	0.13	0.30	0.49	0.24	0.51	0.78	0.62	1.33	2.13
	AUT	−0.54	−1.08	−1.61	−0.64	−1.23	−1.79	−0.81	−1.61	−2.44
	CHE	1.11	2.31	3.65	0.83	1.70	2.67	0.85	1.69	2.55
	NMP	0.07	0.17	0.31	−0.11	−0.19	−0.22	−0.27	−0.52	−0.78
	IRO	0.29	0.71	1.32	−0.61	−1.07	−1.34	−2.14	−4.22	−6.34

**Table 9:** Changes in output by industry according to the increase in the share of wind power generation (% changes in output from the base year).

Scenarios		WIN-LT			WIN-ET			WIN-CT		
Share of Wind Generation (%)		3.0	5.0	7.0	3.0	5.0	7.0	3.0	5.0	7.0
Industries	AFF	−0.34	−0.68	−1.06	−0.28	−0.56	−0.87	−0.24	−0.49	−0.77
	PPP	−0.01	−0.02	−0.01	−0.26	−0.45	−0.61	−0.06	−0.13	−0.19
	MIN	−0.01	0.04	0.16	−0.30	−0.45	−0.53	−0.46	−0.81	−1.15
	FOO	−0.32	−0.64	−1.00	−0.30	−0.58	−0.89	−0.24	−0.50	−0.79
	CLO	−0.24	−0.46	−0.69	−0.39	−0.70	−1.01	0.29	0.49	0.68
	CON	0.00	−0.01	−0.01	−0.01	−0.01	−0.02	−0.01	−0.02	−0.02
	TRN	−0.14	−0.26	−0.39	−0.11	−0.20	−0.30	−0.78	−1.50	−2.31
	SER	−0.14	−0.28	−0.44	−0.15	−0.29	−0.45	−0.13	−0.27	−0.42
	MAC	1.75	3.25	4.90	1.54	2.80	4.14	1.38	2.54	3.72
	ECT	0.45	0.86	1.30	0.62	1.14	1.66	1.26	2.41	3.66
	AUT	−0.71	−1.27	−1.85	−0.86	−1.48	−2.07	−1.14	−2.07	−3.01
	CHE	0.41	0.80	1.26	−0.02	0.01	0.10	−0.02	−0.10	−0.22
	NMP	0.16	0.33	0.54	−0.13	−0.20	−0.22	−0.39	−0.70	−1.00
	IRO	1.00	1.97	3.19	−0.49	−0.71	−0.68	−2.97	−5.32	−7.58

**Table 10:** Changes in total power generation and fossil fuel generation due to the increased solar PV generation (% changes from the base year).

Scenarios	SOL-LT			SOL-ET			SOL-CT		
Share of solar PV generation (%)	3.0	5.0	7.0	3.0	5.0	7.0	3.0	5.0	7.0
Total power generation	0.57	1.39	2.63	−1.28	−2.28	−2.92	−0.35	−0.59	−0.65
Coal power generation	−3.95	−8.32	−13.57	−5.63	−12.25	−20.46	−7.96	−15.60	−23.63
Gas power generation	−14.41	−25.27	−33.94	−18.15	−31.09	−40.17	−12.18	−21.63	−29.99

**Table 11:** Changes in total power generation and fossil fuel generation due to the increased wind generation (% changes from the base year).

Scenarios	WIN-LT			WIN-ET			WIN-CT		
Share of wind generation (%)	3.0	5.0	7.0	3.0	5.0	7.0	3.0	5.0	7.0
Total power generation	0.71	1.55	2.81	−2.29	−3.77	−4.81	−0.82	−1.40	−1.80
Coal power generation	−4.78	−9.10	−14.30	−7.68	−15.11	−24.25	−11.21	−19.92	−28.48
Gas power generation	−16.84	−26.82	−34.90	−22.65	−34.71	−42.58	−13.27	−21.94	−29.80

**Table 12:** Macroeconomic effects of solar PV generation expansion with emission regulations (% changes from the base year).

Scenarios	Indexes	Share of Solar PV Generation (%)			
		1.0 ^a^	3.0	5.0	7.0
SOL-LT/ETS	Welfare	−0.51	−0.61	−0.80	−1.06
	GDP	−0.54	−0.55	−0.60	−0.67
	Wage rate	−1.95	−1.67	−1.34	−0.96
	Electricity price	12.97	10.31	7.00	2.81
	Price of carbon credit (thousand KRW) ^b^	42.57	37.63	32.55	27.42
SOL-ET/ETS	Welfare	−0.51	−0.61	−0.81	−1.05
	GDP	−0.54	−0.56	−0.61	−0.69
	Wage rate	−1.95	−1.74	−1.51	−1.22
	Electricity price	12.97	12.79	12.42	11.12
	Price of carbon credit (thousand KRW) ^b^	42.57	36.46	29.86	22.82

Note: ^a^ This column indicates the situation when the ETS is implemented without renewable expansion policy. ^b^ Indicates the level of price after the equilibrium (not % change from the base year).

**Table 13:** Macroeconomic effects of wind generation expansion with emission regulations (% changes from the base year).

Scenarios	Indexes	Share of Wind Generation (%)			
		0.3 ^a^	3.0	5.0	7.0
WIN-LT/ETS	Welfare	−0.51	−0.81	−1.15	−1.55
	GDP	−0.54	−0.63	−0.75	−0.90
	Wage rate	−1.95	−1.59	−1.29	−0.93
	Electricity price	12.97	9.39	6.08	1.87
	Price of carbon credit (thousand KRW) ^b^	42.57	36.08	31.27	26.36
WIN-ET/ETS	Welfare	−0.51	−0.81	−1.14	−1.52
	GDP	−0.54	−0.65	−0.78	−0.92
	Wage rate	−1.95	−1.74	−1.56	−1.31
	Electricity price	12.97	14.15	14.80	14.23
	Price of carbon credit (thousand KRW) ^b^	42.57	33.82	26.92	19.47

Note: ^a^ This column indicates the situation when the ETS is implemented without renewable expansion policy. ^b^ Indicates the level of price after the equilibrium (not % change from the base year).

**Table 14:** Changes in output by industry according to the increase in the share of solar pv generation with emission regulations (% changes in output from the base year).

Scenarios		ETS Only ^a^	SOL-LT/ETS			SOL-ET/ETS		
Share of Solar PV Generation (%)		1.0	3.0	5.0	7.0	3.0	5.0	7.0
Industries	AFF	0.23	0.10	−0.10	−0.34	0.11	−0.07	−0.30
	PPP	−0.42	−0.36	−0.28	−0.19	−0.46	−0.51	−0.54
	MIN	−2.10	−1.97	−1.79	−1.54	−2.04	−1.93	−1.72
	FOO	0.10	0.00	−0.18	−0.39	−0.01	−0.18	−0.39
	CLO	2.17	1.91	1.61	1.29	1.77	1.29	0.77
	CON	−0.03	−0.03	−0.03	−0.02	−0.03	−0.03	−0.03
	TRN	−3.83	−3.46	−3.09	−2.73	−3.35	−2.85	−2.31
	SER	−0.06	−0.09	−0.14	−0.22	−0.10	−0.16	−0.24
	MAC	−1.45	−1.20	−0.89	−0.53	−1.23	−0.96	−0.62
	ECT	4.38	4.05	3.75	3.45	4.02	3.67	3.26
	AUT	−2.21	−2.46	−2.76	−3.06	−2.48	−2.79	−3.04
	CHE	−2.90	−1.64	−0.15	1.49	−1.77	−0.45	1.00
	NMP	−2.58	−2.29	−1.96	−1.56	−2.35	−2.08	−1.72
	IRO	−16.25	−14.86	−13.22	−11.26	−14.96	−13.39	−11.35

Note: ^a^ This column indicates the situation when the ETS is implemented without renewable expansion policy.

**Table 15:** Changes in output by industry according to the increase in the share of wind generation with emission regulations (% changes in output from the base year).

Scenarios		ETS Only ^a^	WIN-LT/ETS			WIN-ET/ETS		
Share of Wind Generation (%)		0.3	3.0	5.0	7.0	3.0	5.0	7.0
Industries	AFF	0.23	−0.07	−0.38	−0.74	−0.04	−0.33	−0.67
	PPP	−0.42	−0.36	−0.32	−0.25	−0.56	−0.68	−0.77
	MIN	−2.10	−1.89	−1.68	−1.38	−2.03	−1.89	−1.64
	FOO	0.10	−0.17	−0.45	−0.78	−0.17	−0.44	−0.77
	CLO	2.17	1.71	1.31	0.89	1.43	0.80	0.14
	CON	−0.03	−0.03	−0.03	−0.03	−0.03	−0.03	−0.03
	TRN	−3.83	−3.36	−3.04	−2.72	−3.16	−2.64	−2.08
	SER	−0.06	−0.17	−0.29	−0.43	−0.18	−0.31	−0.45
	MAC	−1.45	0.29	1.82	3.52	0.19	1.61	3.17
	ECT	4.38	4.21	4.17	4.14	4.15	4.01	3.79
	AUT	−2.21	−2.58	−2.91	−3.24	−2.63	−2.94	−3.18
	CHE	−2.90	−2.07	−1.37	−0.57	−2.30	−1.79	−1.18
	NMP	−2.58	−2.13	−1.74	−1.28	−2.24	−1.94	−1.52
	IRO	−16.25	−13.88	−11.80	−9.33	−14.07	−12.08	−9.48

Note: ^a^ This column indicates the situation when the ETS is implemented without renewable expansion policy.

**Table 16:** Changes in the total fossil fuel generation source due to the increased solar PV power generation when there are emission regulations (% changes from the base year).

Scenarios	ETS Only ^a^	SOL-LT/ETS			SOL-ET/ETS		
Share of solar PV generation (%)	1.0	3.0	5.0	7.0	3.0	5.0	7.0
Total power generation	−6.63	−5.78	−4.61	−2.97	−6.79	−6.85	−6.52
Coal power generation	−33.43	−31.99	−31.07	−31.08	−31.75	−31.00	−32.13
Gas power generation	18.90	2.39	−13.29	−26.68	−1.25	−20.26	−35.21

Note: ^a^ This column indicates the situation when the ETS is implemented without renewable expansion policy.

**Table 17:** Changes in the total fossil fuel generation source due to the increased wind power generation when there are emission regulations (% changes from the base year).

Scenarios	ETS Only ^a^	WIN-LT/ETS			WIN-ET/ETS		
Share of wind generation (%)	0.3	3.0	5.0	7.0	3.0	5.0	7.0
Total power generation	−6.63	−5.48	−4.29	−2.61	−7.40	−7.85	−7.80
Coal power generation	−33.43	−31.61	−30.93	−31.15	−31.28	−31.16	−33.43
Gas power generation	18.90	−2.42	−16.68	−28.87	−9.09	−26.98	−39.78

Note: ^a^ This column indicates the situation when the ETS is implemented without renewable expansion policy.
